# Protein Phosphatase 2A in the Regulation of Wnt Signaling, Stem Cells, and Cancer

**DOI:** 10.3390/genes9030121

**Published:** 2018-02-26

**Authors:** Joshua J. Thompson, Christopher S. Williams

**Affiliations:** 1 Medical Scientist Training Program, Vanderbilt University, Nashville, TN 37232 USA; joshua.j.thompson@vanderbilt.edu; 2 Department of Medicine, Division of Gastroenterology, Vanderbilt University, Nashville, TN 37232, USA; 3 Veterans Affairs Tennessee Valley Healthcare System, Nashville, TN 37212, USA; 4 Vanderbilt-Ingram Cancer Center, Nashville, TN 37232, USA

**Keywords:** protein phosphatases, Wnt-signaling, stem cells, cancer

## Abstract

Protein phosphorylation is a ubiquitous cellular process that allows for the nuanced and reversible regulation of protein activity. Protein phosphatase 2A (PP2A) is a heterotrimeric serine-threonine phosphatase—composed of a structural, regulatory, and catalytic subunit—that controls a variety of cellular events via protein dephosphorylation. While much is known about PP2A and its basic biochemistry, the diversity of its components—especially the multitude of regulatory subunits—has impeded the determination of PP2A function. As a consequence of this complexity, PP2A has been shown to both positively and negatively regulate signaling networks such as the Wnt pathway. Wnt signaling modulates major developmental processes, and is a dominant mediator of stem cell self-renewal, cell fate, and cancer stem cells. Because PP2A affects Wnt signaling both positively and negatively and at multiple levels, further understanding of this complex dynamic may ultimately provide insight into stem cell biology and how to better treat cancers that result from alterations in Wnt signaling. This review will summarize literature that implicates PP2A as a tumor suppressor, explore PP2A mutations identified in human malignancy, and focus on PP2A in the regulation of Wnt signaling and stem cells so as to better understand how aberrancy in this pathway can contribute to tumorigenesis.

## 1. Introduction

**Figure 1:**
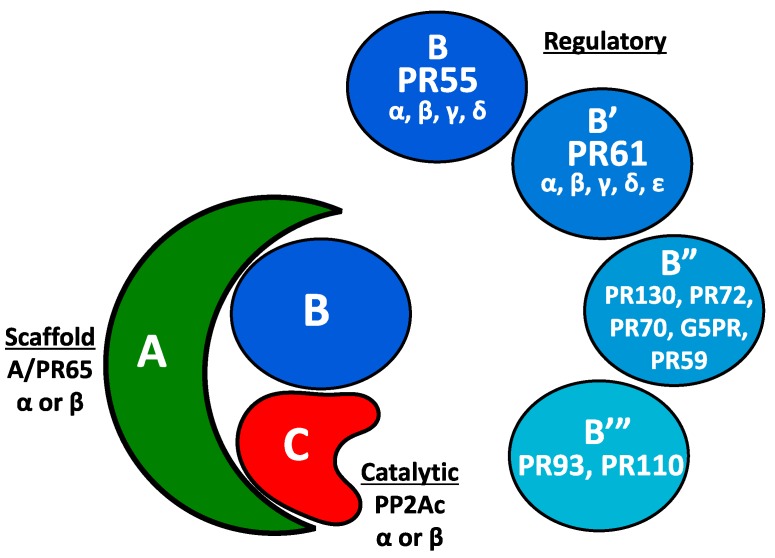
Serine-threonine protein phosphatase 2A (PP2A) holoenzyme. The PP2A holoenzyme consists of a scaffolding (A), regulatory (B), and catalytic (C) subunits. There are two unique scaffolding subunits (PP2A Aα and PP2A Aβ), and two unique catalytic subunits (PP2Acα and PP2Acβ). The regulatory (B) subunits consist of four diverse families: B or PR55, B’ or PR56/PR61, B”, and B’”. Within the B and B’ regulatory subunit families are multiple isoforms, denoted using Greek letters. A number scheme using approximate molecular weights (i.e., PR55 or PR56) is also commonly utilized for referencing PP2A subunits.

Protein phosphorylation is an essential regulator of many cellular processes, including metabolism, transcription, proliferation, cell motility, and apoptosis [1,2]. Nearly 30% of all human proteins are covalently bound to a phosphate—a feat made possible by the 500+ different protein kinases encoded by the human genome [3]. Protein phosphatases make these modifications reversible, and the serine-threonine protein phosphatase 2A (PP2A) accounts for 30–50% of these protein dephosphorylation events [4,5]. PP2A is a heterotrimeric protein complex consisting of a structural (A), a regulatory (B), and a catalytic subunit (C) [6]. There are two unique scaffolding isoforms (Aα and Aβ), two unique catalytic subunit isoforms (cα and cβ), and four structurally diverse families of regulatory (B) subunits that are referred to by a variety of naming conventions: B (or PR55), B’ (PR56/61), B” (PR72/130), and B’” (PR93/110). As shown in Figure 1, Greek letters further identify individual regulatory subunit isoforms of the B and B’ family. These subunits determine the substrate specificity and subcellular localization of PP2A heterotrimers [4,7]. The precision with which PP2A regulatory subunits target individual phospho-residues was established by early studies on the phosphorylation of Simian virus 40 (SV40) large T antigen. A holoenzyme with a B/PR55 family regulatory subunit dephosphorylates Thr124 of the SV40 large T antigen, while a PP2A complex with the B”/PR72 regulatory subunit dephosphorylates Ser120 and Ser123 [8]. The targeting specificity of the regulatory subunits allows a small pool of protein phosphatases to regulate numerous phosphoproteins with enhanced precision [6]. While individual regulatory subunits provide precision, the diversity of subunits also allows for the regulation of a variety of substrates. 

**Figure 2:**
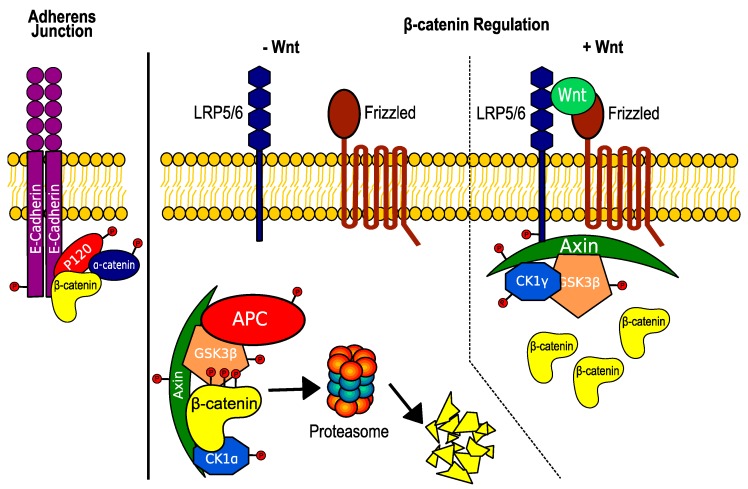
Phosphorylatable proteins involved in the regulation of β-catenin. **Left**: The adherens junction, consisting of E-cadherin, P120, and the catenins, sequesters β-catenin at the plasma membrane. Phosphorylation of E-cadherin at Ser834, Ser836, and Ser842 enhances β-catenin binding affinity, while phosphorylation at Ser846 reduces β-catenin binding. **Middle**: In the absence of Wnt stimulation, β-catenin is bound to the β-catenin destruction complex. CK1α phosphorylation of β-catenin at Ser45 primes β-catenin for subsequent phosphorylation by GSK3β at Ser33, Ser37, and Thr41, which targets β-catenin for proteasomal degradation. Phosphorylation of Axin improves its stability and subsequent ability to negatively regulate Wnt signaling. Axin and APC also contain phosphorylation sites that improve binding to β-catenin. **Right**: In the presence of Wnt ligand, CK1γ phosphorylates LRP5/6, which sequesters Axin at the plasma membrane and prevents the destruction complex from phosphorylating β-catenin. CK1α: casein kinase 1 alpha; GSK3β: glycogen synthase kinase 3 beta; APC: adenomatous polyposis coli; CK1γ: casein kinase 1 gamma; LRP: low-density lipoprotein receptor-related protein.

Wnt signaling is known to regulate patterning and cell fate decisions during embryonic development, and has been implicated in the pathogenesis of cancer [9]. Over 90% of colorectal carcinomas have alterations in Wnt signaling; mutations in the adenomatous polyposis coli (APC) tumor suppressor or activating mutations in β-catenin account for ~80% of cases [10]. The key effector of canonical Wnt signaling, β-catenin, is tightly regulated within the cell, predominantly through two distinct complexes: the adherens junction complex and the β-catenin destruction complex (Figure 2). The adherens junction helps to initiate and stabilize cell–cell adhesion by coupling the transmembrane glycoprotein E-cadherin and associated cytoplasmic catenins with the actin cytoskeleton [11]. E-cadherin can recruit β-catenin to the cell membrane, thereby preventing its nuclear localization [12] in a cell–cell contact-dependent fashion [13]. Cytoplasmic pools of β-catenin are also regulated through the β-catenin destruction complex. In the absence of Wnt ligand stimulation, the cytoplasmic β-catenin destruction complex (composed of the scaffolding proteins Axin and APC and the protein kinases glycogen synthase kinase 3 (GSK3) and casein kinase 1 alpha (CK1α)) binds and phosphorylates β-catenin. This leads to its ubiquitinylation by the beta-transducin repeats-containing protein (β-TrCP) ubiquitin ligase and subsequent proteasomal degradation. CK1α phosphorylation of Ser45 on β-catenin primes the protein for subsequent phosphorylation by GSK3 at Ser33, Ser37, and Thr41, which are required for β-TrCP recognition and β-catenin ubiquitination [14]. In the presence of Wnt ligand (e.g., Wnt3a), Axin is sequestered at the membrane, which prevents assembly of the destruction complex, stabilizes β-catenin [15,16], and allows its translocation to the nucleus for transcription of TCF/β-catenin target genes. Many components of the Wnt pathway can be modified via phosphorylation: the G protein-coupled Wnt receptor Frizzled [17], the Frizzled binding protein Dishevelled [18], the Frizzled co-receptor low-density lipoprotein receptor-related protein-6 (LRP6), components of the β-catenin destruction complex (APC [19,20], Axin [21,22], CK1 [23,24], and GSK3 [25,26]), and β-catenin [14] (Figure 2). Determining the Wnt components targeted by PP2A may identify novel regulatory mechanisms and opportunities for therapeutic intervention.

## 2. A Tumor Suppressive Role for Protein Phosphatase 2A

Early studies using okadaic acid—a serine-threonine phosphatase inhibitor that targets the catalytic PP2Ac subunit—increased tumor formation in a cutaneous carcinogenesis challenge and provided early biochemical support for a tumor suppressive role of PP2A [27,28]. However, this model may suffer from off-target effects as PP2A is inhibited at low doses of okadaic acid [29,30], but increasing concentrations can inhibit multiple protein phosphatases [31]. Subsequent work has more specifically identified a role for PP2A in tumor suppression. The SV40 small T (ST) antigen, the gene product of two transforming DNA viruses—SV40 and polyoma virus—was found to interact with PP2A A and C subunits through co-immunoprecipitation experiments, likely inhibiting PP2A function through displacement of the regulatory subunits [32,33]. Human embryonic kidney cells expressing the catalytic subunit of telomerase, a G12V mutant H-ras, and the SV40 large T antigen (which inactivates the retinoblastoma (RB) and p53 tumor suppressors [34])—otherwise known as HEK TER cells—are immortalized but not tumorigenic (i.e., they lack anchorage-independent growth in soft agar and cannot form tumors in immunocompromised mice [35,36]). However, the addition of ST—which interferes with PP2A function—imparts cells with anchorage-independent growth and the ability to grow as subcutaneous xenografts [36]. Chen et al. determined that this phenotype partially depended upon the B’ regulatory subunit PR61γ-isoform 3 (PR61γ3), as small interfering RNA (siRNA) knockdown of PR61γ3 increased cell proliferation and conferred cells with the ability to grow in soft agar and form tumors in nude mice. Furthermore, overexpression of the regulatory subunit rescued the phenotype, partially reversing tumorigenicity in HEK TER cells as well as human lung cancer cell lines [37]. However, the HEK TER cells with PR61γ3 knockdown formed fewer tumors than HEK TER cells expressing ST, suggesting additional tumor promoting effects of ST aside from just preventing PR61γ3 from incorporating into the PP2A complex. Finally, knockdown of the PP2A Aα scaffolding subunit activates AKT signaling and imparts tumorigenicity to HEK TER cells in immunocompromised mice [38].

Clinical evidence further supports a role of PP2A in tumor suppression. Cancer-associated mutations in the PP2A Aα scaffolding subunit impair binding to specific B subunits as well as the catalytic Cα subunit [39]. Mutations in PP2A Aα appear to act in a dominant negative fashion on wild-type Aα, and also decrease B and C subunit stability, suggesting that an intact PP2A complex stabilizes individual holoenzyme subunits [38]. Additionally, mutations in the PP2A Aβ subunit have been found in human colon cancer, lung cancer, and breast cancer specimens. A list of reported mutations is presented in Table S1. While the majority of these mutations appear to affect the binding of subunits and holoenzyme formation [39], the functional consequences on Wnt signaling have yet to be determined. Clinically, the PP2A inhibitor SET is increased in human non-small cell lung cancer, and leads to poorer overall survival rates, further supporting a tumor suppressive role for PP2A [40]. Additional endogenous inhibitors of PP2A, such as I_1_^PP2A^ (PHAP), may also be clinically relevant; however, additional studies in cancer are needed [41,42]. Reciprocally, small-molecule activators of PP2A (SMAPs) provide a promising avenue for tumor suppression via augmenting PP2A function. KRAS-mutant lung cancer cell lines and xenografts treated with SMAPs lead to the inhibition of tumor growth and apoptosis with reductions in phosphorylated ERK [43]. The PP2A-activating drug FTY720 has also shown promising results in multiple hematologic malignancies [44,45,46]. It is unclear how effective these activators will be in the context of cancers harboring mutations in PP2A, and given PP2A’s dual role in regulating Wnt signaling, these activators may also have dichotomous effects.

## 3. Protein Phosphatase 2A Regulation of E-Cadherin and β-Catenin at the Membrane

Two PP2A catalytic subunits, cα and cβ, share 97% sequence homology [47], yet mice lacking cα die at embryonic day 6.5, demonstrating that cβ cannot compensate for loss of cα [48]. cβ localizes to the cytoplasm and nucleus, while cα is predominantly present at the plasma membrane [49,50]. Thus, subcellular localization may prevent cβ from compensating for loss of cα. Furthermore, β-catenin colocalizes with cα at the plasma membrane in the inner cell mass of early mouse embryos, and loss of cα results in E-cadherin and β-catenin redistribution to the cytoplasm [50]. Destabilization of membrane-bound β-catenin reduces β-catenin levels, likely due to the action of a functional β-catenin destruction complex. While this leads to reductions in total cellular β-catenin levels, the remaining β-catenin is no longer sequestered at the membrane, and is thus free to translocate to the nucleus and induce transcription of β-catenin target genes [12]. Presumably, this makes the cells more responsive to Wnt stimulation. A similar phenomenon is observed in RKO cells, which have a mutation in E-cadherin and low levels of cytoplasmic β-catenin [51] but are exquisitely sensitive to Wnt ligand. In a more recent study, Su et al. demonstrate that PP2Acα knockdown similarly leads to dramatic reductions in membrane-associated and total levels of both β-catenin and E-cadherin in HT29, SW480, DLD1, and HEK293 cell lines [52].

The question of how PP2Acα loss alters E-cadherin localization remains unsolved, but E-cadherin is highly phosphorylated within a serine-enriched domain that comprises the β-catenin binding domain [53]. Phosphorylation of serine residues Ser834, Ser836, and Ser842 enhance β-catenin binding affinity over 300-fold [54,55]. Conversely, CK1-mediated phosphorylation of E-cadherin at Ser846 reduces β-catenin binding and leads to increased E-cadherin internalization [56]. It is plausible that a PP2A complex with a yet-to-be-defined regulatory subunit may specifically dephosphorylate Ser846 on E-cadherin, and that loss of PP2Acα abrogates this interaction, leading to reduced β-catenin binding and E-cadherin internalization.

## 4. Protein Phosphatase 2A’s Dual Regulation of Wnt Signaling in the Cytoplasm

### 4.1. Negative Regulation of Wnt Signaling

PP2A is unlikely to exert its Wnt-inhibitory effects through direct dephosphorylation of β-catenin, as β-catenin dephosphorylation at Ser33, Ser37, and Thr41 removes β-TrCP recognition sites and subsequently stabilizes the protein [14]. Consequently, studies have focused on understanding how PP2A affects other proteins involved in β-catenin regulation. Yokoyama et al. demonstrate that PP2A inhibition via treatment with okadaic acid, knockdown of the PP2Ac catalytic subunit, or treatment with SV40 ST antigen potentiates Wnt signaling following Wnt3a stimulation. Furthermore, all three modifications led to increases in phosphorylated-GSK3β [57]. Phosphorylation of GSK3β at Ser9 suppresses GSK3β kinase activity [25,26], and PP2A treatment can reverse this kinase activity in vitro [58]. Mitra et al. confirm this finding and show that PP2A-mediated dephosphorylation of GSK3β occurs through recruitment of two heatshock proteins: DNAJB6 (DnaJ homolog subfamily B member 6) and HSPA8 (heat-shock cognate protein, HSC70) [59]. An intriguing report focusing specifically on PP2Acα knockout in cardiomyocytes may further elucidate tissue-specific roles of individual PP2Ac isoforms [60]. These data support a potential role of PP2A in dephosphorylating, and thereby activating, GSK3β with resultant phosphorylation of β-catenin leading to its destruction.

Treatment with ST antigen potentiates Wnt signaling and also disrupts B subunit binding to the holoenzyme [31], suggesting that these regulatory subunits aid in inhibiting Wnt signaling. Seeling et al. show that overexpression of the B’ regulatory subunits PR61α, PR61β, PR61δ, PR61ε, and PR61γ3 all decrease exogenous β-catenin in HEK293 cells [61], although an effect on endogenous β-catenin was not evaluated. This decrease in β-catenin was inhibited with okadaic acid treatment, a non-degradable β-catenin mutant that lacks the GSK3β phosphorylation sites, and with proteasomal inhibition, which taken together suggests that PR61-dependent decreases in β-catenin are due to alterations in phosphorylation-induced proteasomal degradation or impaired targeting of β-catenin through the proteasomal degradation pathway. A yeast two-hybrid screen also identified that the PR61α and PR61δ subunits interact with the N-terminal third of APC (unpublished data referenced in [61]) which brings the subunits in close proximity to phospho-residues on Axin, APC, and GSK3β. Overexpression of PR61α in the colorectal cancer HCA7 cell line (wild-type APC) but not the SW480 cell line (APC truncation at 1338) recapitulates the decreases in β-catenin observed in HEK293 cells. These data suggest that PR61α promotes β-catenin degradation through an APC-dependent signaling complex.

Axis duplication experiments in *Xenopus* embryos reveal that the PP2A A, PP2Ac, and B’ PR61α regulatory subunit all have ventralizing activity, indicating Wnt inhibition. In *Xenopus*, β-catenin levels are higher dorsally than ventrally, and higher Wnt tone leads to dorsalization/secondary axis formation [62]. Ventral injection of Wnt agonists leads to secondary body axis formation. To determine where in the Wnt pathway the PP2A regulatory subunits are inhibiting Wnt signaling, epistasis studies using lithium chloride (a GSK3β inhibitor that leads to dorsalization), dominant-negative Axin, and degradation-resistant β-catenin provide evidence that PR61α acts downstream of GSK3β and Axin but upstream of β-catenin to negatively regulate Wnt signaling. Furthermore, PP2A A, PP2Ac, and PR61α co-immunoprecipitate with Axin in *Xenopus* egg extracts, supporting a role for PP2A as a component of the β-catenin degradation complex [63]. Adding to the complexity, another group shortly thereafter demonstrated that two additional B’ family regulatory subunits (PR61β and PR61γ) directly interact with Axin in COS cells. PR61β expression reduces Wnt reporter activity, but did not decrease endogenous β-catenin levels in wild-type L cells, suggesting that PR61β inhibits Wnt signaling through a mechanism independent of β-catenin stability [64]. Taken together, these data highlight the ability of various PP2A components and specifically the regulatory subunits to negatively regulate Wnt signaling at multiple levels.

### 4.2. Positive Regulation of Wnt Signaling

For every piece of evidence that PP2A negatively regulates Wnt signaling, there is evidence to the contrary. Teleological thinking would support a positive role for PP2A in regulating Wnt signaling, as dephosphorylation of the main effector (β-catenin) increases its abundance [14]. Accordingly, Zhang et al. were the first to show that a B family regulatory subunit, PR55α, can interact with β-catenin [65]. Knockdown of PR55α increases β-catenin phosphorylation at Ser33, Ser37, and Thr41 (required for β-TrCP recognition and ubiquitination) in SW480 cells, and also decreases β-catenin levels in HEK293 cells. PR55α overexpression increases Wnt reporter activity in HEK293T cells. Interestingly, phosphorylation of Ser675 (promotes β-catenin stability [66]) and Ser552 (causes β-catenin dissociation from cell–cell contacts and cytosolic/nuclear accumulation [67]) were also increased in SW480 cells with PR55α knockdown. While increased β-catenin stability due to reduced phosphorylation at Ser33, Ser37, and Thr41 appear to trump any effects of Ser675 and Ser552 phosphorylation, the dichotomy highlights the complexity of Wnt phosphorylation and the need for precise phosphatase activity. Hein et al. demonstrated similar results in CD-18/HPAF pancreatic cancer cells, where knockdown of PR55α increased phosphorylation of β-catenin at Ser33, Ser37, and Thr41, destabilized the protein, and reduced total levels of β-catenin. PR55α was increased in human pancreatic ductal adenocarcinoma tissue when compared to normal pancreatic tissue, suggesting that its elevated expression may maintain Wnt signaling and other oncogenic signaling cascades [68].

The scaffolding protein APC is also a putative target of PP2A dephosphorylation and subsequent Wnt activation, as GSK3 phosphorylation of APC improves its ability to bind β-catenin [19,20]. A number of theories exist as to how APC regulates β-catenin levels. APC may promote the export of nuclear β-catenin [69,70], or it may simply sequester β-catenin in the cytoplasm and prevent association with transcription factor 4 (TCF4) in the nucleus [71]. The observation that APC truncations in human colorectal cancers increase total β-catenin levels suggests that APC has a direct role in β-catenin degradation. Su et al. support this hypothesis with evidence that wild type (WT) APC “protects” phosphorylated β-catenin from dephosphorylation by a PP2AAα/PP2Acα dimer, which ensures that the β-TrCP ubiquitin ligase binding site remains intact [52]. This PP2AAα/PP2Acα dimer stabilizes β-catenin by dephosphorylating Ser33 and Ser37, thereby removing the β-TrCP ubiquitin ligase binding site. Mutations in APC abrogate this protective mechanism and allow the PP2AAα/PP2Acα dimer to dephosphorylate β-catenin, shunting it away from the ubiquitination pathway. It should be noted that the PP2Aα/PP2Acα complex identified in this study was isolated from bovine cardiac muscle and utilized in a cell-free system which may limit in vivo correlation. However, this study does highlight the potentially promiscuous nature of PP2A in the absence of a regulatory subunit.

The scaffolding protein Axin has binding sites for both GSK3β and β-catenin, and acts as a negative regulator of Wnt signaling by promoting β-catenin phosphorylation. Axin phosphorylation within the β-catenin binding domain increases binding to β-catenin, stabilizing Axin and increasing β-catenin degradation [22]. Using a combination of yeast-two hybrid screening and co-immunoprecipitation, Hsu et al. showed that Axin can bind directly to the PP2Ac catalytic subunit and mapped this interaction between amino acids 632 and 836 of Axin. Interestingly, this PP2Ac-Axin binding domain is in close proximity to both the GSK3β binding domain (amino acids 477–561) and β-catenin binding domain (amino acids 561–630) [72], again placing phosphatase activity within proximity of putative phosphorylation targets. Using a Wnt reporter assay, Strovel et al. showed that PP2Ac overexpression activates Wnt signaling and that PP2A likely mediates these effects through dephosphorylation of Axin, but the exact target of PP2Ac dephosphorylation has not been determined [73]. Taken together, these data suggest that PP2Ac-mediated dephosphorylation of Axin activates the Wnt pathway.

## 5. Protein Phosphatase 2A Regulation of Stem Cells and Self-Renewal

A role for Wnt signaling in the control of stem cells and cancer stem cells has been well established and previously reviewed [15,74,75,76,77], and the literature reviewed above supports an indirect role of PP2A in regulating stem cells through its modulation of Wnt signaling, yet few studies have looked at Wnt-independent regulation of stemness and self-renewal by PP2A. Wang et al. show that PP2A mediates the equilibrium between self-renewal and differentiation of neural stem cells predominately through regulation of asymmetric division of neural stem cells [78]. Additionally, human embryonic stem cell (hESC) self-renewal has been linked to PP2A activity as forced expression of PP2A reduced levels of SSEA-4, a marker of undifferentiated hESCs [79]. Accordingly, inactivating PP2A via treatment with okadaic acid maintained hESC even in the absence of basic fibroblast growth factor (bFGF)—a key factor known to maintain hESCs [80]. Mechanistically, PP2A inhibition leads to increased phosphorylation of AKT, GSK3β, and Ser62-cMyc with reduced levels of Thr58-phosphorylated c-Myc [79]. Phosphorylation of c-Myc at these two key residues—Thr58 and Ser62—differentially affects c-Myc stability. Phosphorylation at Ser62 stabilizes c-Myc, while phosphorylation on Thr58 signals c-Myc for degradation [81]. PR61α directs PP2A to c-Myc doubly phosphorylated at Thr58/Ser62 and dephosphorylates Ser62, thereby increasing levels of phospho-Thr58 c-Myc. This shift in phosphorylation status signals c-Myc to be degraded by the proteasome [81]. A recent study by Janghorban et al. utilizing a PP2A-PR61α hypomorph mouse with very low levels of PR61α demonstrated hyperproliferation of the epidermis, hair follicles, and sebaceous glands with increased levels of c-Myc phosphorylation at Ser62. Furthermore, PR61α deficiency increased the number of bromodeoxyuridine (BrdU) long-term label-retaining skin stem cells in these mice and enhanced keratinocyte colony formation [82]. Additional studies have indicated c-Myc—a known Wnt target gene [83]—as a regulator of stem cell self-renewal [84,85], highlighting the interplay and complexity of Wnt, c-Myc, and phosphatase signaling. Together, these data support a role for PP2A-PR61α-mediated regulation of stem cell self-renewal and proliferation which may in large part be driven via PP2A mediated c-Myc de-phosphorylation and subsequent stabilization.

## 6. Conclusions

Protein dephosphorylation is a complex and nuanced process, and the PP2A family of serine-threonine phosphatases play an important role in regulating multiple signaling pathways implicated in tumorigenesis, stem cell maintenance, and self-renewal. Early studies of PP2A inhibitors and genomic studies identifying mutations in PP2A subunits support its tumor suppressive role. While perturbations in Wnt signaling can help initiate a number of human malignancies, Wnt signaling is also critical for the maintenance of normal tissue and stem cell homeostasis in the non-transformed state. Ample data supports a role for PP2A as a negative regulator of Wnt signaling; however, there is similarly strong data supporting PP2A’s role in potentiating Wnt signaling. PP2A-mediated regulation of Wnt signaling is likely cellular context-specific, and care must be taken to control for these variables. Given the specificity with which each regulatory subunit targets PP2A-mediated dephosphorylation, future studies must continue to identify how individual trimeric complexes function in regulating a target of interest—referring simply to PP2A provides scant biological relevance. Knockdown and overexpression studies must consider compensatory mechanisms, given the high similarity, yet extreme specificity, of individual regulatory subunits and the numerous levels at which they appear to modulate Wnt signaling. Taken together, the PP2A family of serine/threonine phosphatases regulate Wnt signaling and stemness at multiple levels, both positively and negatively, and further understanding of this complex dynamic will aid in identifying key regulators of tumorigenesis and normal tissue homeostasis.

## Supplementary Materials

The following are available online at http://www.mdpi.com/2073-4425/9/3/121/s1. Table S1: Mutations identified in protein phosphatase 2A (PP2A) scaffolding subunits.
