# Supplementary material for: Protein Phosphatase 2A in the Regulation of Wnt Signaling, Stem Cells, and Cancer
Source: Genes (Basel). 2018 Feb 26;9(3):121. doi: 10.3390/genes9030121 (PMC5867842; doi:10.3390/genes9030121)
Supplement: Supplementary file 1 [file genes-09-00121-s001.pdf]

Table S1. Mutations identified in protein phosphatase 2A (PP2A) scaffolding subunits.

Subunit (Gene)	Tumor Type	Frequency	Mutation	Functional Consequence
**Aα (PPP2R1A)**	Melanoma [1]	1/14 (7%)	Arg-418-Trp	Defective binding to A and/or c subunits [2]. Decreased B regulatory subunit and cα stability [3]
	Lung Cancer [1]	1/16 (6%)	Glu-64-Asp	
	Breast Cancer [1]	2/43 (7%)	Glu-64-Gly<br>Frameshift at AA170	
	MCF-7 breast cancer cell line [4]		Decreased expression	
	Gliomas [5]	25/58 (43%)	Decreased expression	
	Ovarian cancer [6]	3/42 (7%)	Arg-183-Gly<br>Arg-183-Trp<br>Arg-182-Trp	Mutations predicted to impair subunit binding
**Aβ (PPP2R1B)**	Lung Cancer [1]	2/16 (13%)	Exon 9 deletion	
	Breast Cancer [1]	4/43 (9%)	Exon deletion/abnormal splicing	
	Colorectal Cancers	4/30 (13%) [7]	Glyc-15-Ala & Leu-499-Iso Val-498-Glu<br>Val-500-Gly<br>Ser-365-Pro	Mutations predicted to impair subunit binding
		2/13 (15%) [8]		
		5/50 (10%) [9]	Missense: 4/50 (8%)<br>Homozygous deletions: 1/50 (2%)	
	Lung Cancer [8]	5/33 (15%) – Primary<br>4/70 (6%) cell lines	Deletions, frameshifts, and point mutations	Decreased affinity for PP2Ac

References

1. Calin, G.A.; di Iasio, M.G.; Caprini, E.; Vorechovsky, I.; Natali, P.G.; Sozzi, G.; Croce, C.M.; Barbanti-Brodano, G.; Russo, G.; Negrini, M. Low frequency of alterations of the alpha (PPP2R1A) and beta (PPP2R1B) isoforms of the subunit a of the serine-threonine phosphatase 2A in human neoplasms. *Oncogene* **2000**, *19*, 1191–1195.
2. Ruediger, R.; Pham, H.T.; Walter, G. Disruption of protein phosphatase 2A subunit interaction in human cancers with mutations in the A α subunit gene. *Oncogene* **2001**, *20*, 10–15.
3. Chen, W.; Arroyo, J.D.; Timmons, J.C.; Possemato, R.; Hahn, W.C. Cancer-associated PP2A aα subunits induce functional haploinsufficiency and tumorigenicity. *Cancer Res.* **2005**, *65*, 8183–8192.
4. Suzuki, K.; Takahashi, K. Reduced expression of the regulatory A subunit of serine/threonine protein phosphatase 2A in human breast cancer MCF-7 cells. *Int. J. Oncol* **2003**, *23*, 1263–1268.
5. Colella, S.; Ohgaki, H.; Ruediger, R.; Yang, F.; Nakamura, M.; Fujisawa, H.; Kleihues, P.; Walter, G. Reduced expression of the Aα subunit of protein phosphatase 2A in human gliomas in the absence of mutations in the Aα and Aβ subunit genes. *Int. J. Cancer* **2001**, *93*, 798–804.
6. Jones, S.; Wang, T.L.; Shih Ie, M.; Mao, T.L.; Nakayama, K.; Roden, R.; Glas, R.; Slamon, D.; Diaz, L.A., Jr.; Vogelstein, B., et al. Frequent mutations of chromatin remodeling gene *ARID1A* in ovarian clear cell carcinoma. *Science* **2010**, *330*, 228–231.
7. Takagi, Y.; Futamura, M.; Yamaguchi, K.; Aoki, S.; Takahashi, T.; Saji, S. Alterations of the *PPP2R1B* gene located at 11q23 in human colorectal cancers. *Gut* **2000**, *47*, 268–271.
8. Wang, S.S.; Esplin, E.D.; Li, J.L.; Huang, L.; Gazdar, A.; Minna, J.; Evans, G.A. Alterations of the PPP2R1B gene in human lung and colon cancer. *Science* **1998**, *282*, 284–287.
9. Tamaki, M.; Goi, T.; Hirono, Y.; Katayama, K.; Yamaguchi, A. PPP2R1B gene alterations inhibit interaction of PP2A-Abeta and PP2A-c proteins in colorectal cancers. *Oncol. Rep.* **2004**, *11*, 655–659.

© 2018 by the authors. Licensee MDPI, Basel, Switzerland. This article is an open access article distributed under the terms and conditions of the Creative Commons Attribution (CC BY) license (http://creativecommons.org/licenses/by/4.0/).
